# From origins to legacy: the fruits of Canadian medical education scholarship

**Published:** 2019-03-13

**Authors:** Jennifer O’Brien

**Affiliations:** 1 University of Saskatchewan, Saskatchewan, Canada

It is an honour to provide an editorial to introduce the 10^th^ volume of the Canadian Medical Education Journal. Due in no small part to the trailblazers who envisioned this scholarly journal, we at the CMEJ can share the work that is happening in and relevant to medical education in Canada. In the opening paragraphs of the inaugural editorial for volume 1, Dr. Claudio Violate and Dr. Tyrone Donnon wrote,

We are proud to bring you the inaugural issue of the Canadian Medical Education Journal. While we embarked on this adventure with some trepidation a couple of years ago, we are very pleased to launch this initiative. The trepidation has come from the many challenges of starting a new journal. These include establishing an editorial board, the need for high quality submissions of manuscripts, seeking help from expert peer reviewers, and the work required for manuscript selection, preparation and distribution of the issues of the journal as well as the need for resources, time and finances. Notwithstanding, we are pleased to present the first issue of the CMEJ.^1^

In many ways, we face similar challenges in our tenth year to those faced at inception: an evolving and expanded editorial board, the steadily multiplying number of submissions, the ongoing need for expert reviewers, and the demands on editors to keep moving it all forward. Future goals for the CMEJ include indexing in major scholarly databases, establishing a management board, growing our readership, and continuing to work with our partners to expand the scope and depth of medical education in Canada and the journal that supports this. Those of us who roll up our sleeves now are building upon the work of those who laid this foundation. This hard work has paid off. Our editorial board is active and committed. Our partners across the country include the Canadian Association of Medical Education (CAME) Foundation and the Association of Faculties of Medicine of Canada (AFMC), and we will be publishing the abstracts from the CCME this spring. We believe the quality of our work is improving. The CMEJ acceptance rate has declined steadily from 67% in 2010, to 46% in 2015 and 18% in 2018. Even while the number of submissions has multiplied nearly ten-fold between 2014-2018, our peer review process is faster, and by publishing four issues per year instead of two, the time it takes to get articles to publication has also fallen. In this issue, we gratefully acknowledge the contributions of our dedicated reviewers to this success.

In response to these challenges, we are anticipating our big things for CMEJ in the coming months. First, Our Chief Editor Dr. Marcel D’Eon will offer a pre-conference workshop at CCME titled “Writing and reviewing for medical education journals: the CMEJ experience.” Register as a reviewer with the CMEJ and participate in a community of practice. Learn what it takes to publish in the CMEJ! Through a multitude of actions large and small, we continue to refine operations and strive to meet new goals. We expect many fruits of these labours to bear fruit in the coming year. Stayed tuned!

The work of scholars and leaders in the Canadian medical education community exemplifies a commitment to the important task of knowledge translation, or praxis. Praxis, “the act of engaging, applying, exercising, realizing, or practicing ideas,”^2^ describes the process of making things happen. It’s a practical concept, grounded in tangible outcomes. Because I believe in the importance of applied knowledge, I am humbled to serve the CMEJ as the editor of its newest section, *You Should Try This*! Knowledge translation, within a pragmatic paradigm that focuses on ‘what works,’^3^ sparked my interest. The articles in this issue investigate answers to important questions that stem from problems in practice and demonstrate common sense approaches to challenges in medical education. Medical educators – in collaboration with policy makers, learners, educators, and patients – have our work laid out ahead of us.

In the first issue of volume 10, we offer a large selection of articles: eight major contributions, two brief reports, three *Canadiana*, two *You Should Try This!* reports highlighting practical application of theory, a commentary, a book review, and two images.

First, Kobza and colleagues offer lessons learned during implementation of a Canadian diabetes curriculum at the Ottawa Shanghai Joint School of Medicine (OSJSM).^4^ They asked a practical question: “is a Canadian diabetes curriculum suitable for the Chinese population?” While they found that the Canadian curriculum was relevant in China, the authors highlight the areas of need in diabetes education and clinical practice, suggesting a greater emphasis on prediabetes screening in the OSJSM curriculum.

Raman, Lukmanji, Walker, Myhre, Coderre and McLaughlin answer the question of whether the new MCAT can predict licensing exam performance of students in the Cumming School of Medicine at the University of Calgary. They found that MCAT subscores for the biological sciences were predictive of success on the Medical Council of Canada Qualifying Examination (MCCQE) Part 1.^5^ The authors suggest that the MCAT should remain a priority for consideration in admission selections.

Liora Berger and Nishardi Waidyaratne-Wijeratne propose a framework for thinking about wellness, burnout and resiliency in residency training.^6^ These scholars noticed that although previous studies have tried various interventions to reduce resident burnout, the terms resilience and wellness were often conflated. Herein, they outline a framework for fostering resiliency in residents and recommend future work to study the its utility for enhancing the residency experience.

Dagnone et al.^7^ report the implementation of competency based medical education (CBME) at Queen’s over a three-year span. In this illustrative example of praxis, these authors report how one institution took CBME from concept to reality through the development and implementation of a comprehensive strategic plan.

Hall, Mirza, Quinlan, and colleagues – on behalf of Resident Doctors of Canada (RDoC) – tackled the important and persistent problem of resource stewardship in Canadian healthcare delivery.^8^ In collaboration with Choosing Wisely Canada (CWC), they developed an evidence-informed, consensus-based list of five recommendations to promote resource stewardship by Canadian postgraduate trainees.

DeBoer and team, in ‘Building successful and sustainable academic health science partnerships: Exploring perspectives of hospital leaders,’ purposively sampled hospital leaders for their perspectives on AHSPs.^9^ They interviewed 15 individuals in a variety of leadership roles and found six dimensions of influence in the partnership between academic centers and hospitals. This work has important implications for hospital and academic leadership looking to leverage opportunities in their own contexts.

We are excited to share a study from our Finnish colleagues at the University of Turku in this issue of the CMEJ. Vilppu, Laakkonen, Mikkilä-Erdmann, and Kääpä examined the connections between study success and the regulation strategy profiles (the ability to actively monitor and regulate one’s learning by using various cognitive, metacognitive, and behavioural strategies) of medical and dental students over the first 3 years of medical school.^10^ They found that students with an increasing lack of regulation and lowest self-regulation had lower GPAs than other groups, with implications for the work of curriculum developers and medical educators.

Moreau, Eady, and Jabbour’s work is meaningful to the social contract of medicine in the context of competency based medical education.^11^ They asked important questions: to what extent, and in what ways, can we involve patients in resident assessment within a competency framework? They further report the facilitators and hindrances to such involvement, laying important groundwork for implementation of patient engaged CBME. Let this be a call to action for us all!

You will find several other excellent pieces in this tenth issue of the CMEJ, beginning with the inspired art^12,13^ that graces the cover, and including an additional two brief reports,^14^^,15^ three uniquely *Canadiana* contributions,^16^^-18^ two practice-grounded suggestions that *You Should Try This*!^19^^,20^ and two well-rounded recommendations regarding online lectures^21^ and the *Journal of Art and Healing*.^22^

And last (but not least!), we acknowledge the hard work of our dedicated CMEJ reviewers who make it all happen!
